# Changes in Blood DNA CpG Methylation Levels in Response to Methadone Maintenance Treatment: Epigenome-Wide Longitudinal Study

**DOI:** 10.3390/epigenomes10010018

**Published:** 2026-03-05

**Authors:** Orna Levran, Yuli Kim, Justin Li, Anat Sason, Miriam Adelson, Einat Peles

**Affiliations:** 1 The Laboratory of the Biology of Addictive Diseases, The Rockefeller University, New York, NY 10065, USA; yulikim424@gmail.com; 2 Dr. Miriam & Sheldon G. Adelson Clinic for Drug Abuse Treatment & Research, Tel Aviv Sourasky University Medical Center, Tel Aviv 6423906, Israel; anatsas@tlvmc.gov.il (A.S.); miriam.adelson@adfam.com (M.A.); einatp@tlvmc.gov.il (E.P.); 3 AccuraScience LLC, Johnston, IA 50131, USA; justin.t.li@lbs.accurascience.com; 4 Gray Faculty of Medical and Health Sciences, Tel Aviv University, Tel Aviv 6997801, Israel; 5 Sagol School of Neuroscience, Tel Aviv University, Tel Aviv 6997801, Israel

**Keywords:** DNA methylation, longitudinal, MMT, opioid use disorder, *DGLUCY*, *SOX10*, *NPAS3*, oligodendrocytes

## Abstract

Background/Objectives: Methadone maintenance treatment (MMT) is one of the major pharmacotherapies for opioid use disorder. The underlying mechanisms of addiction and the treatment response are only partially understood. The study’s main goal was to identify differential DNA CpG methylation that occurred in response to MMT. Methods: Toward this goal, we have conducted a longitudinal epigenome-wide study of blood samples from 64 patients at the beginning and after 1–3 years of MMT, using a linear mixed model. Results: A total of 1881 differentially methylated probes (DMPs) were identified (FDR < 0.05), controlling for sex, age, estimates of blood cell proportions, and the first two principal components based on genome-wide SNP genotypes. Among the genes annotated to the top DMPs are *DGLUCY*, *NXNL2*, *SOX10*, and *NPAS3*. Several genes associated with substance use disorder were annotated by the identified DMPs, including *ADORA2A*, *BDNF*, *CACNA1D*, *CREB1*, *CRHR1*, *CRY1*, *DNMT3B*, *GABRD*, *GNAS*, *GRIP1*, *OXR1*, *PRKACB*, *SCN2A*, and *SCN3A*. The most overrepresented pathway is the small GTPase-mediated signal transduction pathway, and the most overrepresented process is the actin cytoskeleton organization. Conclusions: The study provides preliminary insight into the epigenetic effect of MMT. Future studies will have to confirm the DMPs, assess their impact on gene expression, and determine their clinical relevance.

## 1. Introduction

Opioid use disorder (OUD) is a public health challenge that is characterized by compulsive seeking and abuse of opioids, regardless of destructive consequences [1,2]. Opioids have an important role in managing chronic pain, and over-prescription has contributed to the opioid epidemic [3]. Recently, there has been an increase in the use of potent synthetic opioids like fentanyl [4]. OUD is caused by a combination of biological and environmental factors. Opioid abuse changes the brain reward circuitry, and these neuroadaptations are mediated in part by epigenetic mechanisms [5]. Several risk variants for OUD have been identified by candidate gene and genome-wide association studies (GWAS); however, they account for a small proportion of the known heritability (e.g., [6]).

Methadone maintenance treatment (MMT) is one of the major pharmacotherapies for treating opioid addiction, in addition to buprenorphine and extended-release naltrexone [7,8]. These medications target the mu-opioid receptor within the endogenous opioid system. MMT is characterized by large interindividual dose variability, and relapse remains a major challenge [9]. The underlying mechanisms of MMT response are only partially understood.

DNA methylation influences the accessibility of the DNA to transcription factors and other regulatory proteins in a tissue-specific manner and dynamically regulates gene expression [10]. It may offer insight into the pathophysiology of a disease and/or serve as a biomarker (e.g., risk, subtype, or therapeutic response). Data on blood DNA methylation and opioid use are limited [11]. Some candidate gene studies showed alterations in blood DNA methylation in individuals with heroin addiction or opioid use. Differential methylation was indicated in the promoters of the mu-opioid receptor gene (*OPRM1*) (e.g., [12]). An epigenome-wide association study (EWAS) of European American women with OUD that used opioid-exposed controls [13] and an EWAS of active injection drug use reported several epigenome-wide significant CpG [14]. Numerous recent review articles provide additional information from preclinical studies and references to specific studies (e.g., [5,6,15]). Several differentially methylated probes (DMPs) were indicated to be associated with the methadone daily dose (for review, see [16]). A meta-analysis identified DMPs related to opioid medication use [17]. DNA methylation quantitative trait loci (meQTLs) are genetic variants that interact with DMPs to modulate gene expression and can be far apart. Several meQTLs in the dopamine receptor D1 gene, *DRD1*, were associated with OUD and the effective dosage of methadone [18].

The cross-sectional design of previous methylation studies from our laboratory [12,19] did not allow the distinction between the effects of OUD and MMT. The current study was designed to allow this distinction by using a longitudinal approach and examining changes in DNA methylation at admission to MMT and after 1–3 years of treatment. Since DNA methylation can be both a cause and a consequence of gene expression changes, longitudinal studies are useful to elucidate the causal relationships between DNA methylation and a specific phenotype. This approach also reduces the effect of potential confounders.

The main goal of the current study was to examine whether MMT for OUD is associated with changes in DNA methylation levels in peripheral blood. These changes may reveal epigenetic processes underlying the response to MMT, serve as biomarkers, or represent side effects of treatment.

## 2. Results

The demographic and clinical characteristics of the samples are shown in Table 1. The study included only subjects of major European and Middle Eastern descent based on principal component analysis (PCA) of genome-wide genotype data (Figure S1).

A total of 1881 CpGs met the criteria for DMP, showing a significant difference in methylation levels between baseline and follow-up samples. Group, age, sex, batch, two population-stratification PCs (from genome-wide genotype data), and six blood cell-type composition estimates were included as covariates (FDR < 0.05, Table S1). About 16% of the DMPs are in proximity to the transcription start site (TSS200 and TSS1500). These DMPs are annotated to 2014 genes. DMPs with a small change (|delta beta| < 0.02) were filtered out to reduce false positives. Delta beta values for the remaining DMPs were generally modest in magnitude, and only a small subset exceeded ±0.05 (Figure S2). DMPs reported to be related to tobacco smoking (based on the EWAS open platform and other studies [20]) were excluded from the final list (Table S1) to reduce ambiguity and are presented in Table S2.

**Figure 1:**
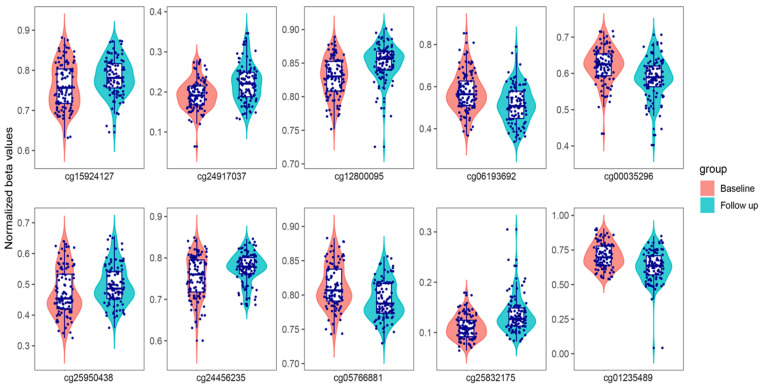
Violin plots of the top differentially methylated probes (DMPs) associated with time at MMT in the longitudinal analysis. The violin plots show the distribution of normalized DNA methylation (beta values). The vertical axis represents the range of values, where 0 and 1 mean completely unmethylated and completely methylated, respectively. The box plot shows the interquartile range of the dataset (25% at the bottom, 75% at the top), and the middle line shows the median.

Table 2 lists the top ten-ranked DMPs, and violin plots from normalized beta values are shown in Figure 1.

Three of the top DMPs are located in regulatory regions, and two of them are located in regulatory regions of transcript variants. CpG cg05766881 is located at the 5′ UTR of an alternative protein-coding transcript (ENST00000698177.1) and upstream of the reference transcript of the SOX10 gene (SRY-box transcription factor 10). CpG cg25832175 is located in a regulatory region, 72 bp upstream of an alternative transcript (ENST00000479731.5) of *FAM107B* (family with sequence similarity 107 member B) that encodes a smaller protein. The region includes binding sites for several transcription factors, including Fos and Jun (OREG1632150). CpG cg24917037 is located in a regulatory region (promoter, CpG island) upstream of the nucleoredoxin-like 2 gene, *NXNL2*, as well as upstream of an lncRNA (ENSG00000301889) in the antisense orientation. NXNL2 has a role in synaptic plasticity. Three of the top DMPs are located in intronic sequences with an enhancer-like signature that may bind regulatory proteins to activate transcription.

It is not yet known whether the DMPs are correlated with gene expression or function, but some of the annotated genes may be of interest. The genes annotated to cg25950438 and cg05766881 (*NPAS3* and *SOX10*, respectively) encode transcription factors that are highly expressed in oligodendrocytes. The gene annotated to cg15924127 (*DGLUCY*) is involved in the metabolism of D-glutamate and D-serine, which are crucial signaling molecules for glutamate receptors. Three of the top DMPs are annotated to genes highly expressed in the testis (*ARGFX* and *USP37*). CpG cg01235489 is located in an intron of *USP37* and appeared to have significant differences in methylation levels between males and females in our sample (delta beta = 0.056). The effect of MMT on the methylation levels was not significantly different between sexes.

Searching the DMPs for differentially methylated regions revealed four adjacent CpGs with a similar direction of the methylation difference in the promoter region of the CD19 molecule gene, *CD19* (cg07597976, cg10571406, cg05433111, and cg01758575).

One of the top-ranked DMPs showed a significant difference of 5.6% between males and females in our cohort. DMP cg01235489 is located within an intron of the ubiquitin-specific peptidase 37 (*USP37*) gene, which is highly expressed in the testis. The effect of MMT on the methylation of cg01235489 was similar in both sexes.

Among the significant DMPs set (*n* = 1881), there are CpGs annotated to genes that are known to be associated with addiction-related phenotypes or encode methadone target proteins, including *ADORA2A*, *BDNF*, *CACNA1D*, *CREB1*, *CRHR1*, *CRY1*, *DNMT3B*, *GABRD*, *GNAS*, *GRIP1*, *OXR1*, *PRKACB*, *SCN2A*, and *SCN3A*. In addition, one DMP is annotated to the promoter of *CYP2C19*, which is related to methadone metabolism.

Gene ontology (GO), Reactome, and KEGG pathway enrichment analyses were conducted to assess the potential biological relevance of the gene set annotated by the DMPs identified in the longitudinal study. These genes were overrepresented in several pathways, including actin cytoskeleton organization and small GTPase-mediated signal transduction (Figure S3).

A comparison of the DMPs identified in the current study with previously published related studies revealed that cg15924127 and cg25950438 were indicated in a previous EWAS of OUD and heroin-exposed controls in women of European ancestry, although they did not reach the set threshold for genome-wide significance in that study [13].

## 3. Discussion

The main goal of the study was to examine whether MMT for OUD is associated with changes in DNA methylation levels in peripheral blood. These changes may reveal epigenetic processes underlying the response to MMT, serve as biomarkers, or represent side effects of treatment. Toward this goal, we have conducted a longitudinal study. To our knowledge, this is the first longitudinal epigenome-wide study of MMT. Although the overall changes in methylation levels identified in the longitudinal study were modest, the study identified several novel targets of interest that warrant further exploration.

One of the main findings of the study is increased methylation levels of cg25950438 at *NPAS3* (neuronal PAS domain protein 3) after more than 1 year of treatment. It is not yet known whether this DMP is correlated with gene expression. This CpG was also indicated in a previous EWAS of OUD in European American women, although it did not reach genome-wide significance [13]. NPAS3 is a bHLH transcription factor that is highly expressed in oligodendrocytes and is implicated in psychiatric and neurodevelopmental disorders [21,22]. Notably, cg25950438 showed high brain–blood correlation in a previous study that used live brain samples from Japanese subjects [23], but a similar study in European subjects did not replicate this finding [24]. The clinical significance of this finding is not clear at this point.

A potentially related finding is the reduced methylation levels of cg05766881 during MMT. This CpG is located at the 5′ UTR of an alternative protein-coding transcript of *SOX10*. SOX10 is highly expressed in oligodendrocytes, and a reduction in oligodendrocyte-specific expression of *Sox10* was observed in the nucleus accumbens following acute morphine treatment in mice [25]. Furthermore, viral overexpression of *Sox10* in the prefrontal cortex was shown to regulate heroin addiction-like behavior in male rats [26].

Oligodendrocytes are a type of glial cell that form the myelin sheath around axons and have a vital role in shaping neural transmission. Myelin plasticity is recognized to be associated with opioid reward and addiction, and myelination is a potential therapeutic target for OUD [27,28]. The role of glial cells in addiction may be related to the actions of drugs as well as to the cell dysregulation caused by relapse and withdrawal [29].

Another potential gene of interest is nucleoredoxin-like 2, *NXNL2*, which is thought to play a role in synaptic plasticity. *Nxnl2* knockout mice showed severe behavioral deficiency in fear, pain sensitivity, coordination, learning, and memory [30]. *DGLUCY* is another potential gene of interest as it is involved in the metabolism of signaling molecules for glutamate receptors. The glutamate system plays a central role in substance addiction [31].

A group of four adjacent DMPs in the promoter region of *CD19* was shown to change methylation levels in the same direction, forming a differentially methylated region (DMR). A similar DMR was reported in a study comparing CD19+ B cells between patients with multiple sclerosis and controls [32]. The functional significance of this DMR is uncertain. CD19 is a biomarker for B cells that is critical for the immune response. Opioid use is linked to the activation of various immune cells and the elevation of biomarkers of chronic inflammation [33]. It was suggested that the immune system abnormalities in patients with heroin addiction can be restored by MMT [34].

DMPs annotated to genes that are known to be associated with addiction-related phenotypes or encode methadone target proteins, including *ADORA2A*, *BDNF*, *CACNA1D*, *CREB1*, *CRHR1*, *CRY1*, *DNMT3B*, *GABRD*, *GNAS*, *GRIP1*, *OXR1*, *PRKACB*, *RTP4*, *SCN2A*, and *SCN3A*, were also identified. Although some of them are located in regulatory regions, further studies are necessary to assess their functionality.

An exploratory pathway enrichment analysis of the gene set annotated by the DMPs was performed to generate hypotheses. Enrichment of the small GTPase-mediated signal transduction pathway and the actin filament organization process was indicated. These findings are interesting since opioid use leads to changes in the expression of GTPases, which cause cytoskeleton remodeling and synaptic changes in dopamine neurons, leading to addiction and relapse [35]. Actin is the primary component of the membrane cytoskeleton, and it interacts with numerous proteins, including small GTPase regulatory proteins like Rho family GTPases. There has been an interest in targeting cytoskeleton dynamics to correct drug-induced brain plasticity changes as therapeutics for substance use disorders. It is possible that MMT changes the gene expression of these GTPases and modulates brain plasticity changes induced by OUD. At this point, it is not yet known whether the small methylation changes identified in the study in peripheral blood cause clinically relevant changes in gene expression.

One of the top-ranked DMPs showed a significant 5.6% difference between males and females in our cohort. DMP cg01235489 is located in an intron of the ubiquitin-specific peptidase 37 (*USP37)* gene, which is highly expressed in the testis.

There are significant sex differences in DNA methylation [36]. Epigenetic aberrations are known to be associated with male infertility [37]. Opioid abuse affects sperm morphology and motility and may lead to male infertility [38]. In previous studies of opioid use for chronic pain, men had significantly lower DNA methylation of selected CpG sites in the *OPRM1* promoter [39]. Semen analyses of patients in MMT showed sperm abnormalities in a smaller percentage of patients compared to OUD patients not in treatment [40]. The current study identified DMPs in several genes that are highly expressed in the testis, including the testis-specific *FAM107A*. *Fam107a* exhibits abnormal morphology and reduced sperm motility in rodents [41]. If the different DNA methylation levels in the blood cause a functional change in gene expression and also reflect the expression of these genes in the testis, it may suggest a mechanism by which heroin abuse and MMT affect male fertility.

As expected, the vast majority of the patients in this study are current or former cigarette smokers [42]. Cigarette smoking induces CpG methylation, and some of these changes may persist for years after cessation [20]. To reduce ambiguity and identify epigenetic changes independent of tobacco-related changes, reported smoking-related CpGs are presented separately.

Several DMPs identified in the current study were previously identified in a cross-sectional EWAS of OUD in women of European ancestry and heroin-exposed controls [13]. These DMPs were highly significant in the previous study but did not reach the set threshold for genome-wide significance. DNA methylation can be both a cause and a consequence of drug abuse. However, EWAS of OUD and controls cannot distinguish between the effects of heroin abuse, genetic predisposition to OUD, or treatment effects. DMPs identified in both the EWAS of OUD and the current study, if they turn out to regulate gene expression, are most probably not associated with a predisposition to OUD.

This study used blood as a surrogate tissue for the brain to study epigenetic changes in the immune system. The CNS–immune system interplay is bidirectional. Peripheral immune cells and peripherally released cytokines can infiltrate the brain and contribute to the activation of glial cells [43]. It is not known what role blood DMPs play in the brain, but they can potentially serve as biomarkers. A small number of CpGs have high brain–blood correlation [23].

Several issues have to be considered when interpreting the results. The current evidence for a relationship between MMT and DNA methylation difference is correlational. It is not yet known whether the DMPs are correlated with gene expression or function. Additionally, many DMPs are not annotated to any gene, and others are annotated to the nearby genes. It cannot be determined at this stage whether these DMPs have therapeutic value, reflecting the attenuation of the OUD effects by MMT, or are a side effect of MMT. Another limitation is that we were unable to obtain most of the blood samples before the start of the treatment, as would have been the optimal design.

A small effect size indicates that the difference occurs in a small fraction of the cells. Nevertheless, the difference could affect the function of these cells and may still have a significant effect on the phenotype. Other studies reported small effect sizes on a similar scale [44]. Other limitations of the study are the sample size and the potential for unaccounted technical and biological confounding factors. Several potential residual confounders could not be incorporated into the analysis without substantially reducing the sample size or model stability ( such as cocaine and benzodiazepine use, as well as comorbidities).

The main finding of this study is that there are modest changes in the methylation levels of numerous CpGs in peripheral blood after 1–3 years of methadone maintenance treatment. It is not known at this point if these DMP cause a functional change in gene expression. Some of these DMPs are annotated to potential genes of interest, including transcription factors, genes involved in synaptic plasticity, small GTPase-mediated signal transduction, actin filament organization, signaling molecules for glutamate receptors, and genes associated with addiction-related phenotypes. Some of the DMPs are located in regulatory regions, and some were indicated in a previous EWAS of OUD.

In conclusion, this exploratory study provides insight into the epigenetic effect of MMT. Future studies will have to corroborate and verify these preliminary findings, assess their effect on gene expression, and determine their clinical relevance.

## 4. Materials and Methods

### 4.1. Study Samples

Patients with OUD were recruited from The Dr. Miriam & Sheldon G. Adelson Clinic for Drug Abuse Treatment & Research, Tel Aviv Sourasky Medical Center. All Individuals had one or more years of daily multiple uses of opioids and at least one withdrawal or failure in a detoxification center (for details, see [45]). Clinical details were collected using the Kreek-McHugh-Schluger-Kellogg (KMSK) scale, an instrument for the assessment of a lifetime diagnosis of opiate, cocaine, alcohol, and other illicit substances [46], the Addiction Severity Index [47], and the Diagnostic and Statistical Manual of Mental Disorders, 4th Edition (DSM-IV).

Only patients of major European and Middle Eastern descent were included based on self-report and principal component analysis PCA (see below). Subjects were included in the study if the blood samples from which their DNA was extracted were drawn at the beginning and up to 3 years in treatment. Subjects were not included in the study if the current MMT was not their first treatment. Subjects were not excluded from the study if they tested positive for heroin during treatment. All the subjects signed informed consent for genetic studies. The study was approved by the institutional review boards of Tel Aviv Sourasky Medical Center (Helsinki Committee-03-240TLV) and Rockefeller University Hospital.

### 4.2. Genome-Wide SNP Genotyping

Genomic DNA was extracted from peripheral whole blood using the Puregene Blood Kit (Qiagen, Germantown, MA, USA). DNA was quantified using a Qubit™ 3.0 fluorometer (Thermo Fisher Scientific, Waltham, MA, USA). Genotyping was conducted using the Illumina Infinium Global Screening Array (GSA-24 v3.0, Illumina, San Diego, CA, USA) at the Icahn Institute for Genomics and Multiscale Biology at Mount Sinai (New York, NY, USA). The reference samples were also genotyped on the Smokescreen^®^ Genotyping Array [48,49]. Data was analyzed using the Genome Studio software genotyping Module v2 (Illumina). The following criteria were also used for quality control: (1) detected SNPs > 99% at the individual level; (2) genotype calling rate > 99% at the SNP level; and (3) a *p*-value for the Hardy–Weinberg equilibrium >1× 10^−6^. Individuals with call rates <98% were excluded. Genotypes were checked for concordance with the data obtained from SNP probes on the EPIC array. The minimum required SNP concordance was 0.99, and the minimum required concordance for a given individual was 0.9.

### 4.3. Principal Component Analysis (PCA)

PLINK (v1.9) was used to conduct PCA using the filtered genome-wide SNP set of all the samples and reference samples from Europe, Africa, Central Asia, and Far East Asia. Extreme outlier samples were removed (Figure S1a). Two PCs explained most of the variability in this sample set (Figure S1b) and were included as covariates in the analyses.

### 4.4. DNA Methylation Profiling

One microgram of DNA was treated with a bisulfite conversion process using the Premium Bisulfite kit (Cat#C02030030) by the epigenomic services of Hologic Diagenode (Diagenode, Seraing, Belgium). DNA methylation (5-methylcytosine) quantification was conducted using the Illumina Infinium Human Methylation EPIC BeadChip array (v1.0 and v2.0) targeting >850,000 genome-wide methylation sites in biologically significant regions (Illumina, San Diego, CA, USA; Cat # G02090000, G0209006, respectively). Methylation levels were calculated as beta values, which describe the percentage of methylated DNA and range from 0 to 1.

### 4.5. Sample Quality Control (QC)

QC was performed based on internal control probes using Genome Studio Software 2011.1, Methylation Module v1.9, as well as the meffil R package (v1.1.1) [50]. QC was conducted independently by the epigenomic services of Hologic Diagenode and AccuraScience LLC (Johnston, IA, USA). The following parameters were checked: sex mismatch, methylated vs. unmethylated signal comparison, detection *p*-value > 0.05, the number of beads per CpG < 3, bisulfite conversion statistics, atypical blood cell-type composition estimates, and lack of concordance between genotypes obtained using the Illumina Infinium v3.0 array and the EPIC array.

### 4.6. Probes Pre-Processing

Only the 721,745 probes that are shared between the two versions of the array (v1.0 and v2.0) were included, implying that probes new to the v2.0 version and probes that were removed by the company in the new version were excluded. Probes were excluded from analysis based on the following criteria: probes mapped to multiple places in the genome, probes mapped to sex chromosomes, and probes with a detection *p*-value > 0.05. A total of 705,484 probes passed QC. Beta values were normalized using “meffil.normalize.samples”. Probes with normalized beta values below 0.05 were excluded. A total of 672,472 CpGs were retained for analysis.

### 4.7. Estimated Blood Cell Subtype Proportions

Assessment of the blood cell-type proportions was conducted using a multiplexed molecular platform of CpG methylation data [51]. The relative proportions of six white blood cell types (i.e., B, T CD8+, T CD4+, Natural killer (NK), Monocytes, and Neutrophils) were estimated. A set of 306 CpGs (out of the original 329 CpGs [51]) was available after filtering. A linear model was constructed with sex, age, and batch (version of EPIC arrays) included as covariates. Adjusted beta values were obtained as residuals of the linear model to account for the effect of the covariates and were rescaled to a range of between 0 and 1. The function “meffil.estimate.cell.counts.from.betas” that estimates cell counts from a reference was used with the rescaled adjusted beta values. An optimized library for reference-based deconvolution was adopted as a reference [52] using the function “blood IDOLoptimized epic”.

### 4.8. Longitudinal Study

A sophisticated linear mixed model was constructed to determine the differential methylation for each CpG site between the 64 DNA samples that were obtained at the beginning of the treatment and the 64 samples that were obtained from the same patients after 1–3 years in MMT (Table 1). In addition, the study included non-repeated DNA samples from a set of 72 individuals that were obtained at similar times to the longitudinal samples, as well as 10 technical replicates.

CpG methylation level was treated as a dependent variable. Group, age, sex, batch, two population stratification PCs (from genome-wide genotype data), and six blood cell-type composition estimates were included as additional independent variables (confounders). The patient was included as a random effect, making the linear model equivalent to a paired test, but including confounders. The model was constructed using the “lmerTest” package (version 3.1-3) in R (version 4.2.1). Beta values were normalized as described. Adjusted beta values were obtained as residuals of the linear model. Combined box plots and violin plots were created with normalized and adjusted beta values using the “ggpubr” R package (version 0.6.0). False discovery rate (FDR) control was conducted using the “p.adjust” function in R with the parameter “method=BH”. The significance threshold was set at q-value < 0.05.

### 4.9. Bioinformatics Analysis

Annotation of DMPs was conducted using the annotation file downloaded from the Illumina EPIC v2.0 resources (https://support.illumina.com/array/array_kits/infinium-methylationepic-beadchip-kit/downloads.html, accessed on 1 September 2025). Annotation of genes was conducted using the current UCSC RefGene annotation file (GENECODE v48). Genes were included only if the distance between the CpG and the transcript start site (TSS) was less than 1500 bp.

The EWAS Open Platform (https://ngdc.cncb.ac.cn/ewas/, accessed on 1 September 2025) was used to identify reported tobacco smoking-related CpGs. Genomic information was obtained from Ensembl, The Genome Aggregation Database (GnomAD), V2.1.1 (https://gnomad.broadinstitute.org/), the University of California, Santa Cruz (UCSC) Genome Browser (http://genome.ucsc.edu/cgi-bin/hgGateway, accessed on 1 September 2025), LDlink (https://ldlink.nih.gov), and The Genotype-Tissue Expression (GTEx) project dataset (https://gtexportal.org).

Gene Ontology (GO) enrichment analysis was conducted on the list of genes corresponding to the significant DMPs using the clusterProfiler (version 4.10.0) package in the R software (version 4.2.1), including biological process (BP) pathways, cellular component (CC), and molecular function (MF). A gene set was obtained from the MSigDB database (https://www.gsea-msigdb.org/gsea/msigdb/index.jsp, accessed on 1 September 2025). Kyoto Encyclopedia of Genes and Genomes (KEGG) pathway enrichment analysis and Reactome pathway enrichment analyses were conducted using the clusterProfiler (version 1.46.0) package in the R software.

## Figures and Tables

**Table 1:** Demographic and clinical data of the sample.

Variable	Baseline	Follow-Up
*n*	64	64
Age range	30–74	32–76
The proportion of females	0.19	0.19
Time to blood sample	<1 month	1–3 years
Methadone dose mg/day (mean)	0–120 (62)	20–215 (126)

**Table 2:** DMPs associated with MMT.

#	DMP	Chr	Position (hg38)	Gene(s)	*p*-Value	FDR	Baseline Beta Mean	Follow-Up Beta Mean	Delta Beta	Region and Regulatory Functions
1	cg15924127	14	91,174,129	*DGLUCY*	4.85 × 10^−8^	0.003	0.76	0.78	0.02	intron, enhancer-like signature
2	cg24917037	9	88,534,956	*NXNL2*; *ENSG00000301889*	8.91 × 10^−8^	0.003	0.19	0.22	0.03	TSS200 (promoter, CpG island)
3	cg12800095	1	46,128,415	*PIK3R3*; *P3R3URF-PIK3R3*	1.07 × 10^−7^	0.003	0.83	0.85	0.02	intron, enhancer-like signature
4	cg06193692	12	85,721,163	interegenic	2.43 × 10^−7^	0.004	0.57	0.51	−0.06	H3K27Ac mark
5	cg00035296	3	121,569,645	*ARGFX*	4.06 × 10^−7^	0.004	0.62	0.59	−0.04	TSS1500 (ENST00000651603.1); intron (ENST00000334384.5)
6	cg25950438	14	33,607,948	*NPAS3*	4.39 × 10^−7^	0.004	0.47	0.50	0.02	intron
7	cg24456235	5	52,856,921	*ITGA1*; *ENSG00000288539*	4.43 × 10^−7^	0.004	0.75	0.78	0.02	intron, enhancer-like signature
8	cg05766881	22	37,985,166	*SOX10*; *POLR2F*	5.17 × 10^−7^	0.004	0.81	0.79	−0.02	5′ UTR alternative transcript (ENST00000698177.1); TSS1500 (ENST00000396884.8); intron
9	cg25832175	10	14,572,386	*FAM107B*	5.79 × 10^−7^	0.004	0.11	0.14	0.03	TSS200 alternative transcript (ENST00000479731.5); intron; H3K27Ac mark
10	cg01235489	2	218,502,346	*USP37*; *RN7SKP38*	5.96 × 10^−7^	0.004	0.71	0.63	−0.07	intron

TSS200: 200 bp from transcription start site; TSS1500: 1500 bp from transcription start site.

## Data Availability

Data are available upon request.

## Supplementary Materials

The following supporting information can be downloaded at https://www.mdpi.com/article/10.3390/epigenomes10010018/s1: Figure S1. Scatter plot of the two main principal components based on genome-wide genotype data. (a) Study samples and references for the main continents. (b) Study samples without references. Each dot represents one individual. Color code: green—the study sample; red—European; blue—Central Asia; purple—Africans; orange—Far Asia; Figure S2. Delta beta values distribution across the DMPs; Figure S3. Gene ontology (GO) biological process (BP) and KEGG pathway enrichment analyses of the longitudinal study significant DMPs (q < 0.05); Table S1. CpGs showing a significant difference in methylation levels between baseline and follow-up samples (FDR < 0.05); Table S2. Smoking-related CpGs showed a significant difference in methylation levels between baseline and follow-up samples (q < 0.05).

## Abbreviations

The following abbreviations are used in this manuscript:

DMP	Differentially methylated probe
OUD	Opioid use disorder
MMT	Methadone maintenance treatment
EWAS	Epigenome-wide association study
meQTLs	Methylation quantitative trait loci
PCA	Principal component analysis
QC	Quality control
GO	Gene ontology
