# Supplementary material for: Changes in Blood DNA CpG Methylation Levels in Response to Methadone Maintenance Treatment: Epigenome-Wide Longitudinal Study
Source: Epigenomes. 2026 Mar 5;10(1):18. doi: 10.3390/epigenomes10010018 (PMC13024794; doi:10.3390/epigenomes10010018)

Figure S1. Scatter plot of the two main principal components based on genome-wide genotype data. a. study sample and references for the main continents. b. study sample without references. Each dot represents one individual. Color code: green: the study sample; red: European; blue: Central Asia; purple: Africans; orange: Far Asia.

a.

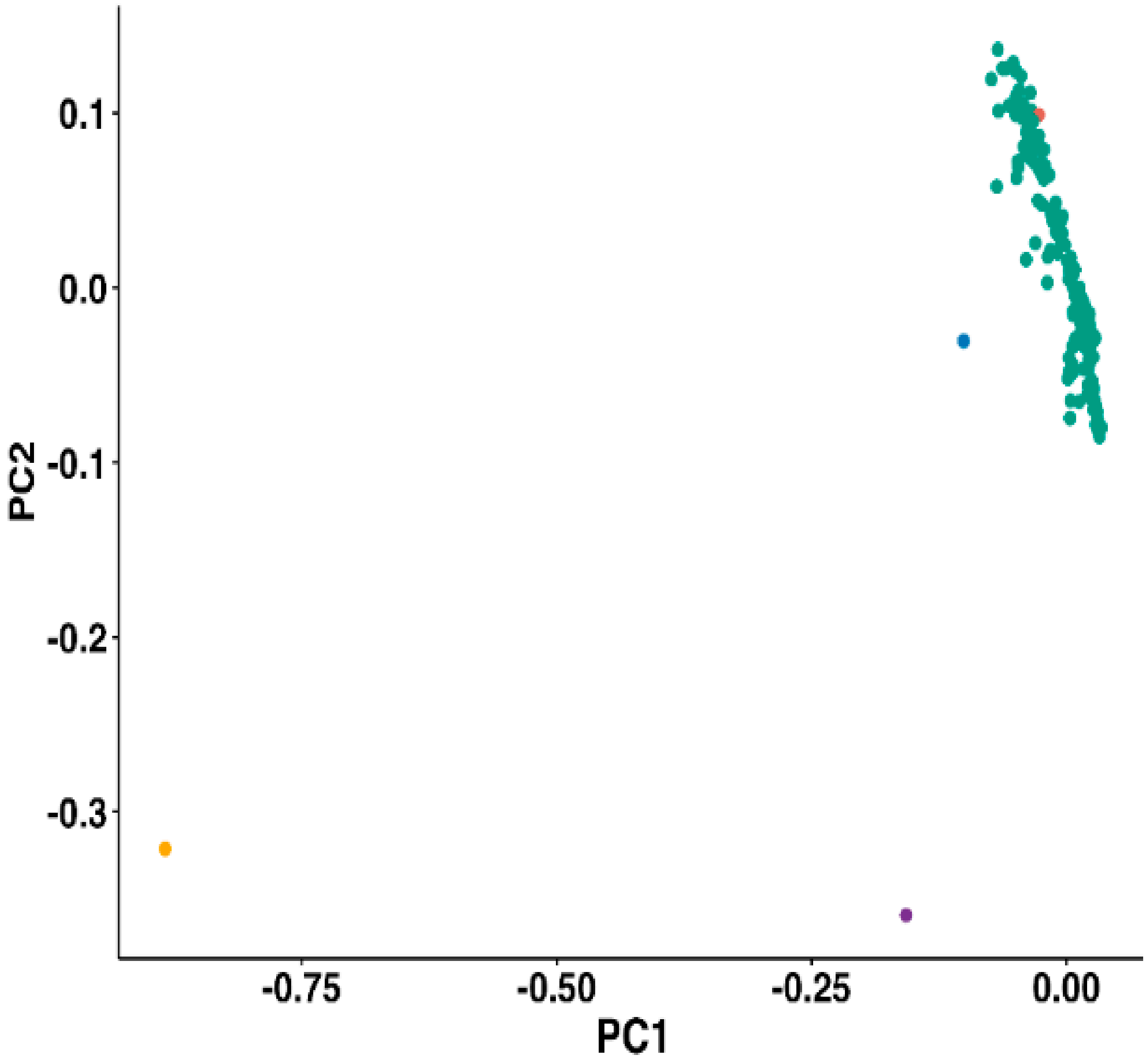

b.

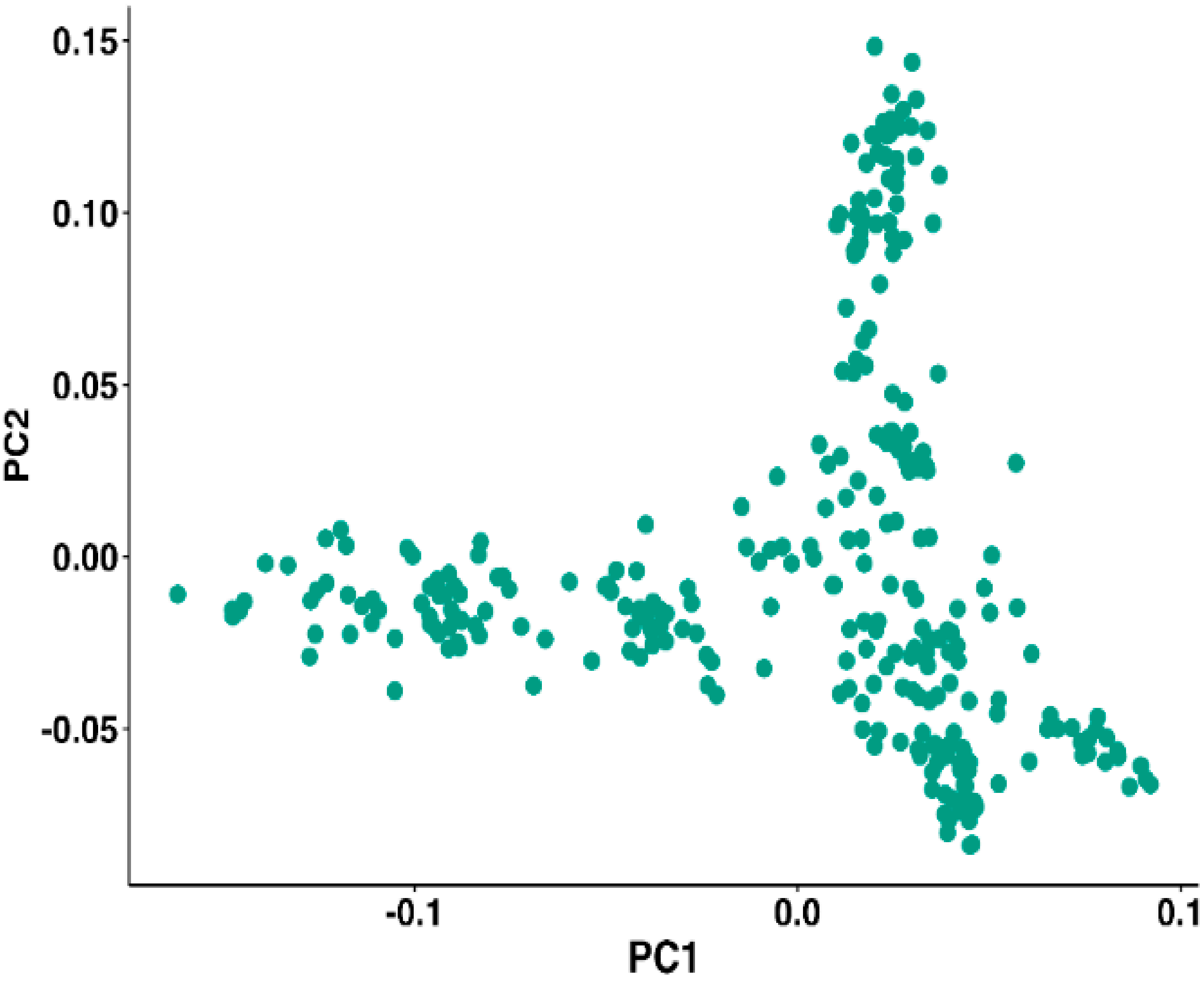

Supplement: Supplementary file 1 [file epigenomes-10-00018-s001.zip › Levran Figure S1 Feb 2026.pdf]
