# Supplementary material for: Changes in Blood DNA CpG Methylation Levels in Response to Methadone Maintenance Treatment: Epigenome-Wide Longitudinal Study
Source: Epigenomes. 2026 Mar 5;10(1):18. doi: 10.3390/epigenomes10010018 (PMC13024794; doi:10.3390/epigenomes10010018)
Supplement: Supplementary file 1 [file epigenomes-10-00018-s001.zip › Levran Figure S3 Feb 2026.pdf]

Figure S3. The top 20 enrichment pathways identified by GO, KEGG, and Reactome analyses of the genes annotated by the significant DMPs (q < 0.05) in the longitudinal study

GO-Biological Pathways

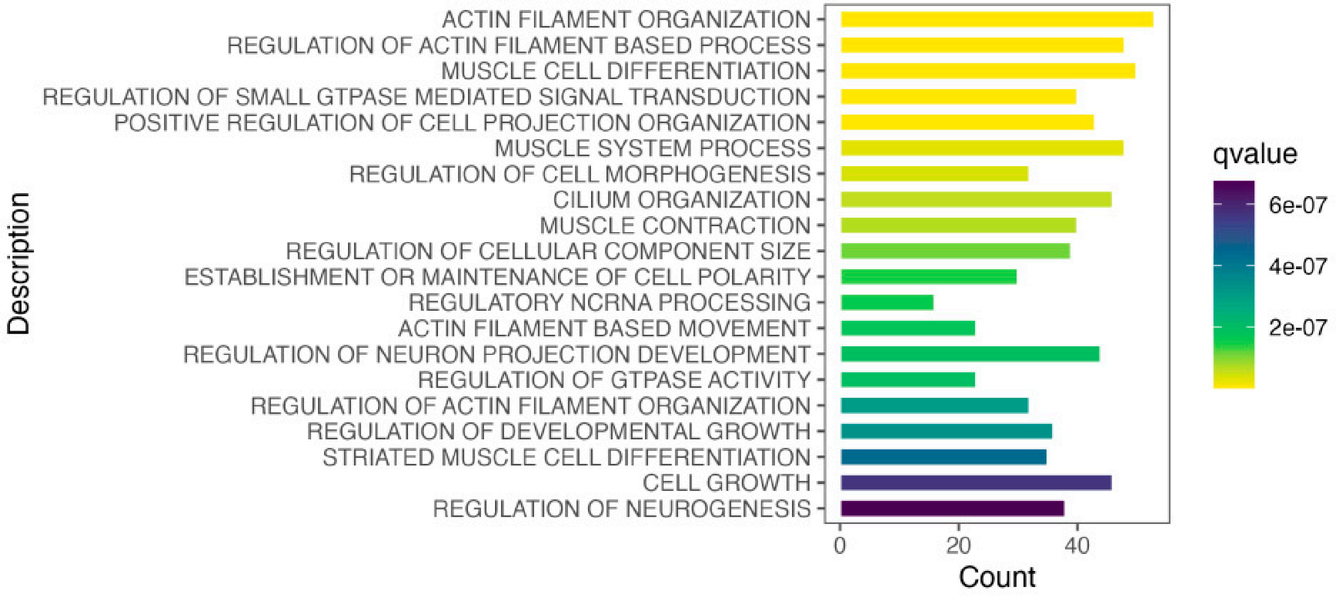

GO- Molecular Function

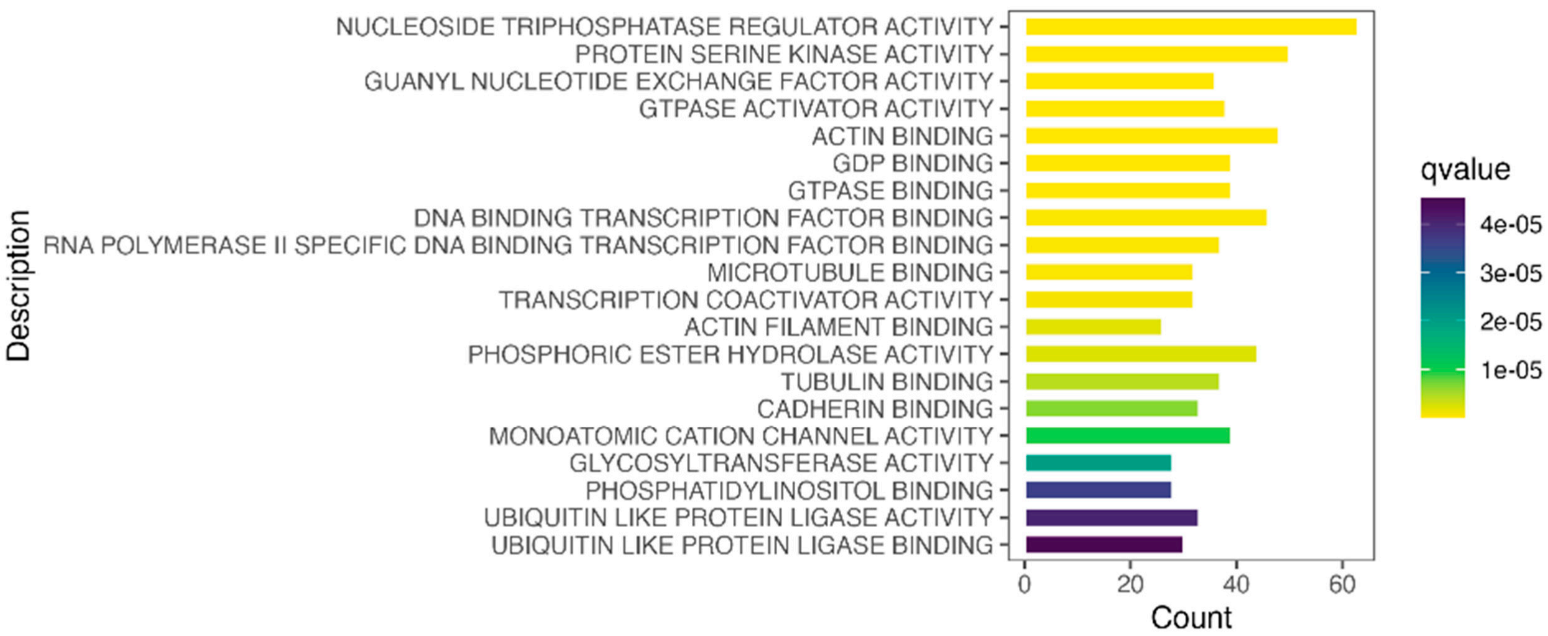

KEGG

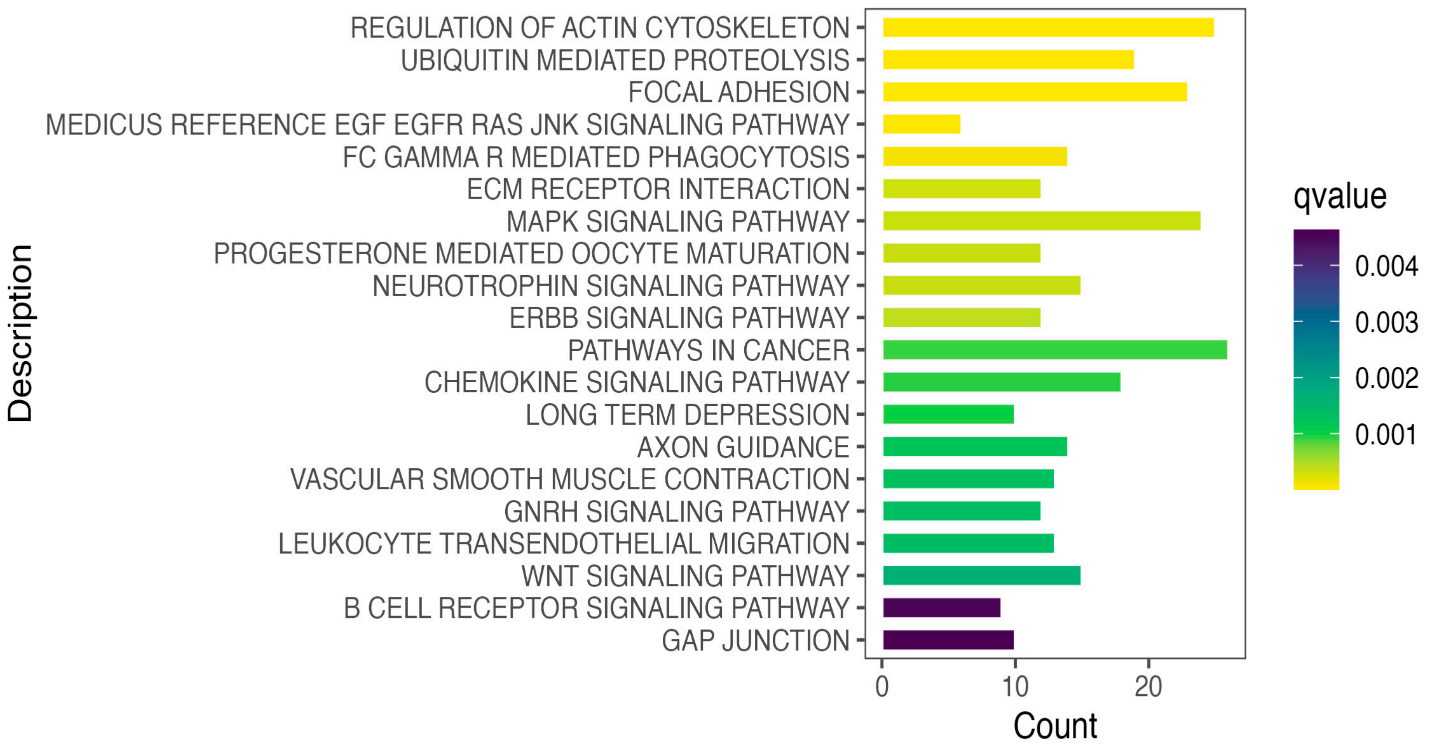

REGULATION OF ACTIN CYTOSKELETON
UBIQUITIN MEDIATED PROTEOLYSIS
FOCAL ADHESION
MEDICUS REFERENCE EGF EGFR RAS JNK SIGNALING PATHWAY
FC GAMMA R MEDIATED PHAGOCYTOSIS
ECM RECEPTOR INTERACTION
MAPK SIGNALING PATHWAY
PROGESTERONE MEDIATED OOCYTE MATURATION
NEUROTROPHIN SIGNALING PATHWAY
ERBB SIGNALING PATHWAY
PATHWAYS IN CANCER
CHEMOKINE SIGNALING PATHWAY
LONG TERM DEPRESSION
AXON GUIDANCE
VASCULAR SMOOTH MUSCLE CONTRACTION
GNRH SIGNALING PATHWAY
LEUKOCYTE TRANSENDOTHELIAL MIGRATION
WNT SIGNALING PATHWAY
B CELL RECEPTOR SIGNALING PATHWAY
GAP JUNCTION
Description
0
10
20
Count
qvalue
0.004
0.003
0.002
0.001

Reactome

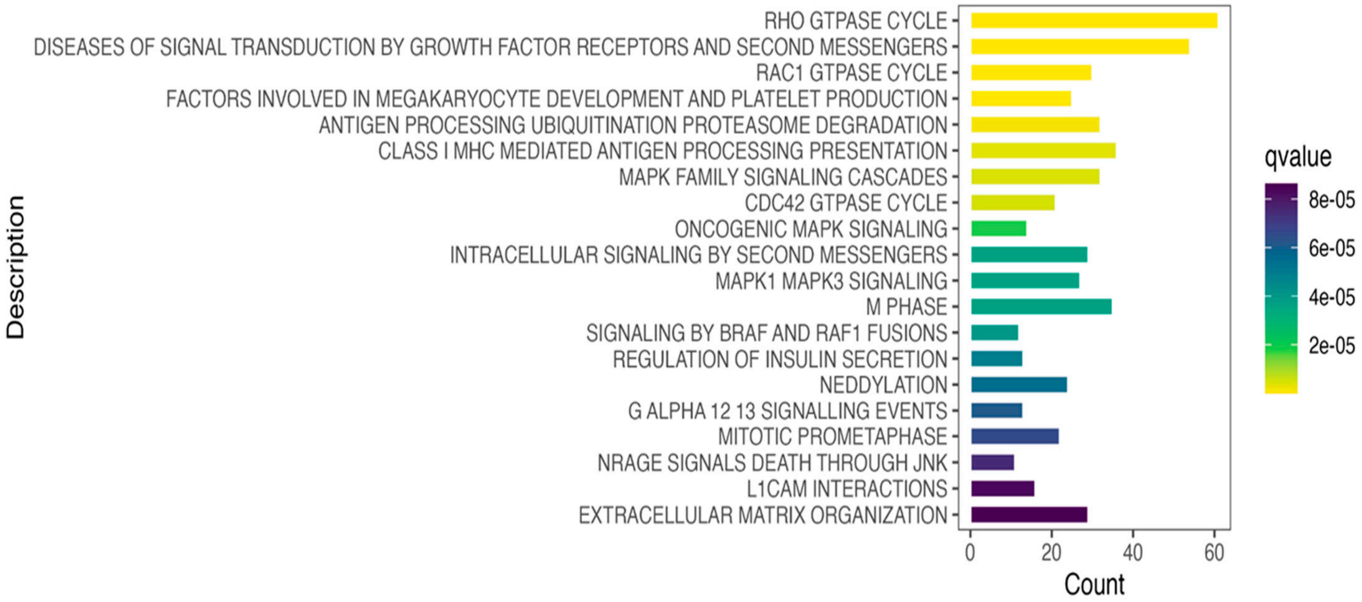

RHO GTPASE CYCLE
DISEASES OF SIGNAL TRANSDUCTION BY GROWTH FACTOR RECEPTORS AND SECOND MESSENGERS
RAC1 GTPASE CYCLE
FACTORS INVOLVED IN MEGAKARYOCYTE DEVELOPMENT AND PLATELET PRODUCTION
ANTIGEN PROCESSING UBIQUITINATION PROTEASOME DEGRADATION
CLASS I MHC MEDIATED ANTIGEN PROCESSING PRESENTATION
MAPK FAMILY SIGNALING CASCADES
CDC42 GTPASE CYCLE
ONCOGENIC MAPK SIGNALING
INTRACELLULAR SIGNALING BY SECOND MESSENGERS
MAPK1 MAPK3 SIGNALING
M PHASE
SIGNALING BY BRAF AND RAF1 FUSIONS
REGULATION OF INSULIN SECRETION
NEDDYLATION
G ALPHA 12 13 SIGNALLING EVENTS
MITOTIC PROMETAPHASE
NRAGE SIGNALS DEATH THROUGH JNK
L1CAM INTERACTIONS
EXTRACELLULAR MATRIX ORGANIZATION
Description
0
20
40
60
Count
qvalue
8e-05
6e-05
4e-05
2e-05
