# Supplementary figures and images for: Changes in Blood DNA CpG Methylation Levels in Response to Methadone Maintenance Treatment: Epigenome-Wide Longitudinal Study
Source: Epigenomes. 2026 Mar 5;10(1):18. doi: 10.3390/epigenomes10010018 (PMC13024794; doi:10.3390/epigenomes10010018)

**Figure S2. Delta beta values distribution across the DMPs**

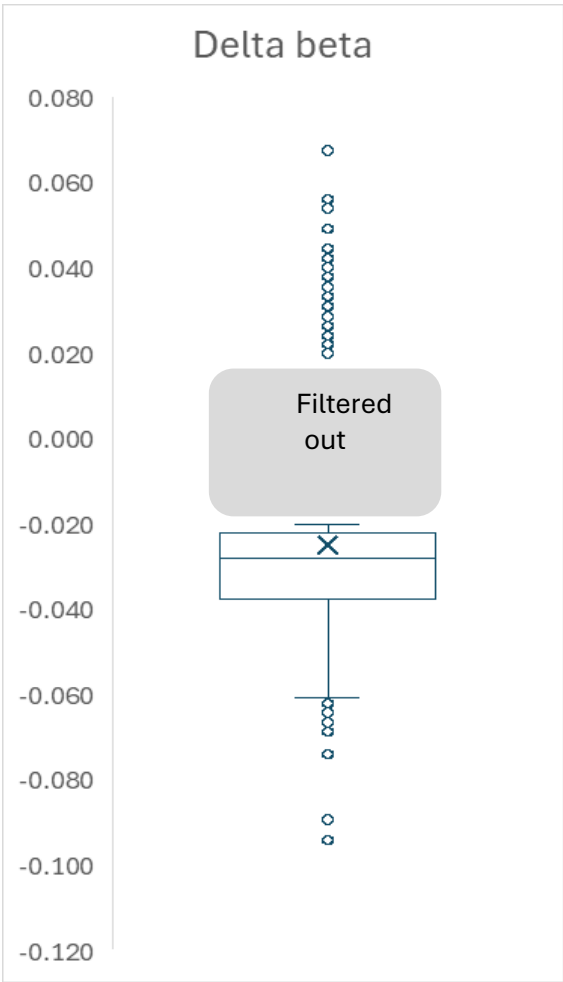

Supplement: Supplementary file 1 [file epigenomes-10-00018-s001.zip › Levran Figure S2 Feb 2026.pdf]
